# Monitoring lymphatic filariasis interventions: Adult mosquito sampling, and improved PCR – based pool screening method for *Wuchereria bancrofti *infection in *Anopheles *mosquitoes

**DOI:** 10.1186/1475-2883-6-13

**Published:** 2007-11-29

**Authors:** Daniel A Boakye, Helena A Baidoo, Evans Glah, Charles Brown, Maxwell Appawu, Michael D Wilson

**Affiliations:** 1 Department of Parasitology, Noguchi Memorial Institute for Medical Research, University of Ghana, P.O. Box LG 581, Legon, Accra, Ghana

## Abstract

**Background:**

Monitoring and evaluation are essential to the successful implementation of mass drug administration programmes for LF elimination. Monitoring transmission when it is low requires both large numbers of mosquito vectors and sensitive methods for detecting *Wuchereria bancrofti *infections in them. PCR-based methods are preferred over classical dissections but the best protocol so far achieved detection of one L_3 _*Wuchereria bancrofti *larva in a pool of 35–50 *Anopheles *mosquitoes. It also lacks consistency and remains still a costly tool. Hence we decided to improve upon this to achieve detection in a pool of 100 or more by enhancing the quality of the template DNA. Prior to this we also evaluated three vector sampling methods in the context of numbers for monitoring.

**Methods:**

Human landing, pyrethrium spray and light traps catches were conducted concurrently at sites in an LF endemic district in Ghana and the numbers obtained compared. Two DNA extraction methods; Bender buffer and phenol/chloroform purification, and DNAeasy Tissue kit (Quaigen Inc) were used on pools of 25, 50, 75 100 and 150 mosquitoes each seeded with one L_3 _or its quivalent amount of DNA. Then another set of extracted DNA by the two methods was subjected to Dynal bead purification method (using capture oligonucleotide primers). These were used as template DNA in PCR to amplify *W. bancrofti *sequences. The best PCR result was then evaluated in the field at five sites by comparing its results (infections per 1000 mosquitoes) with that of dissection of roughly equal samples sizes.

**Results:**

The largest numbers of mosquitoes were obtained with the human landing catches at all the sites sampled. Although PCR detection of one L_3 _in pools of 25, 50 and 75 mosquitoes was consistent irrespective of the extraction method, that of one L_3 _in 100 was only achieved with the kit-extracted DNA/Dynal bead purification method. Infections were found at only two sites by both dissection and pool-screening being 14.3 and 19 versus 13.4 and 20.1 per 1000 *Anopheles *mosquitoes respectively, which were not statistically significant

**Discussion and conclusion:**

HLC still remains the best option for sampling for the large numbers of mosquitoes required for monitoring transmission during MDA programmes, when vector population densities are high and classical indices of transmission are required. One – in – 100 detection is an improvement on previous PCR pool-screening methods, which in our opinion was a result of the introduction of the extra step of parasite DNA capture using Dynal/beads. As pool sizes increase the insects DNA will swamp parasite DNA making the latter less available for an efficient PCR, therefore we propose either additional steps of parasite DNA capture or real-time PCR to improve further the pool screening method. The study also attests also to the applicability of Katholi *et al*'s algorithm developed for determining onchocerciasis prevalence in LF studies.

## Background

Lymphatic filariasis, a debilitating disease affects over 128 million who have either circulating microfilariae or one of the various clinical conditions associated with filarial infection with nearly 1.2 billion people also at risk. Due to the global importance the 50^th ^meeting of the World Health Assembly in May 1997 resolved to eliminate lymphatic filariasis by the year 2020. The adopted strategy is mass drug administration (MDA) with the combination therapy of albendazole/DEC and albendazole/ivermectin for areas where the disease is co-endemic with onchocerciasis. Monitoring of this intervention strategy is an essential component of the elimination programme. This can be achieved either through the surveillance of either microfilaraemia or antigenaemia levels in the community or of infection rates in the vector populations. However, when control measures are instituted the level of infection drops to low levels such that the classical method of dissecting insect vectors to determine infection rates becomes less sensitive and highly labour intensive [1].

Recent technological advances in molecular biology have addressed the shortcomings of traditional methods for parasite detection an example is the use of species-specific oligonucleotide probes and primers in PCR-based assays in *W. bancrofti *diagnosis [2]. The ability to detect *W. bancrofti *DNA in a PCR-based assay more importantly has been exploited for the detection of infected mosquitoes. PCR detection of infections in mosquitoes is much less labour intensive and tedious than dissection. However, the cost of PCR in identifying parasites in each infected fly is prohibitive if large numbers of mosquitoes are to be examined. In such situations modification of the PCR method to detect parasites in a pool of mosquitoes [3] could be cost effective.

This PCR based method has been employed to detect one infective *Wuchereria *larva in a pool of about 14 – 50 *Anopheles *mosquitoes [4,5] and 30 – 50 *Aedes *mosquitoes [6]. The cost effectiveness of the method could be improved if the number of mosquitoes in a pool could be increased whilst maintaining the sensitivity of the technique. In attempts at achieving the above, several laboratories used different protocols and this invariably led to reports of inconsistent results. Similar results were also obtained by a multi-laboratory study to standardise methodologies but the Fischer method was deemed the best [5]. From our assessment of this report we surmised that a major limiting factor with the pool-screening methodology could be the purity of the isolated DNA.

The present study therefore is aimed at revisiting the extraction methods to obtain quality DNA for PCR and to achieve the detection of one infective *W. bancrofti *larva (L_3_) in a pool of 100 or more mosquitoes.

Another anticipated problem associated with monitoring when parasite levels are low is the requirement of large numbers of mosquitoes to detect any infection. Various mosquito sampling methods are used in entomological studies so we evaluated three of them to determine which one will be adequate for monitoring purposes.

## Materials and methods

### Evaluation of mosquito sampling methods

Three standard adult collection methods [7]; human landing catches (HLC), light traps (LTC) and pyrethrum spray catches (PSC) were all used over two days in three villages within the Winneba District of Ghana. The light traps were set at four different locations in the villages

### Human landing catches

Six locals were recruited to catch night-biting mosquitoes from randomly selected compounds. At each compound, three sat indoor and three outdoor from 1800 hrs and 0600 hrs for 50 minutes of the hour. The two teams were rotated between indoor and outdoor after each collection period, to compensate for individual differences in attractiveness. Thus 12 human-night catches were made for each village. The captured mosquitoes were kept in paper cups labeled to denote the hour and location.

### Pyrethrum spray catches

Randomly selected rooms (1 room/house) were sprayed with pyrethroid insecticide formulation (Raid^® ^Insecticide: Tetramethrin, Allethrin, Deltamethrin) and 10 minutes after spraying, the knockdown mosquitoes were picked and placed on moist filter paper in labeled petri dishes. Collections were made in 5 houses per village.

### Light trap

Two suitable rooms located in four ends of the villages were selected for the light trap collection. In each room, a miniature CDC light trap with a standard 6 V 100 mA incandescent bulb and powered by 6 dry cell batteries was hung from1800 h to 0600 h. Thus mosquito collections were made with 8 traps/night for 2 nights

The mosquitoes obtained by each method were sorted according to species using the criteria of Gilles and De Meillon [8] and counted. The mosquitoes were then killed and stored at -40 until needed.

### Evaluation of DNA extraction methods

For this study, fresh adult *An. gambiae *s.l. mosquitoes were reared from larvae and pupae collected from breeding sites within the Accra metropolis. In addition adult mosquitoes were also collected using landing catches at Dodowa and other areas in Ghana known to be non-endemic for LF. The females were sorted from the males and stored at -40°C until needed.

The mosquitoes were divided into pools of 25, 50, 100, 150, and 200 and dried in heating block at 95°C for three hours. The Qiagen DNeasy^® ^kit (Quiagen Inc, Mississauga, Canada) protocol and Bender buffer method of Flook *et al. *[9] for DNA extraction were used. The DNeasy protocol for animal tissue was slightly modified for the pools of mosquitoes. Briefly the mosquito pools were homogenised in 90 μl of Buffer ATL and 10 μl Proteinase K and incubated at 55°C for three hours. Buffering conditions were adjusted with 100 μl of Buffer AL to provide ideal DNA binding conditions and the lysate was loaded onto the DNeasy mini-column membrane. After brief centrifugation, the DNA selectively binds to the DNeasy membrane whilst contaminants pass through. This was followed by two steps of washing to remove any remaining contaminants and enzyme inhibitors and the DNA was then eluted in buffer. The eluted DNA in buffer was mixed with 2× volume of absolute alcohol and 0.25× of 8 M potassium acetate and incubated overnight at 4°C, and centrifuged at 15,000 rpm for 15 minutes. The DNA pellets were washed with 70% ethanol, and air dried. The pellets were then suspended in 20 μl of MilliQ™ water and seeded with DNA extracted from 1 L_3_.

To simulate field conditions, pools of mosquitoes were also homogenised in tubes that contained either one or two L_3_, s. The positive and negative controls were tubes that contained L_3_s only, and pools of mosquitoes with no L_3_, respectively. The Qiagen extraction protocol was used and the extracted DNA treated as described above.

The second DNA extraction method followed Flook *et al. *[9] Briefly the same distribution of mosquito pools and *W. bancrofti *described above were homogenized in Bender buffer (0.1 M NaCl, 0.2 M sucrose, 0.1 M Tris-HCl, 0.05 M EDTA (pH 9.1), 0.5% SDS, in sterile double distilled water). The DNA was isolated by ethanol precipitation followed by two phenol/chloroform purification steps. The DNA pellets were suspended in 20 μl sdd water. All the DNA samples were kept at -40°C until ready to use.

### Dynabead purification and PCR amplification of *W. bancrofti *DNA

The DNA samples extracted using the above two methods were further purified using the manufacturer's protocol for Dynabeads M-280 Streptavidin^® ^(Dynal AS Oslo, Norway). For this, the volume of DNA samples were made up to 100 ul with the DNA binding buffer (100 mM Tris-HCl [pH 7.5], 100 mM Nacl) and 25 nM final concentration of biotinylated *W. bancrofti *capture primer (5' biotin-CC CTC ACT TAC CAT AAG ACA AC) added. The mixture was heated at 95°C for 3 minutes, cooled slowly to 35°C, to allow the annealing of the capture primer to its complement sequence of the parasite DNA. Then 100 μg of streptavidin-coated magnetic beads equilibrated in DNA binding buffer added to each mix. The mixture was incubated overnight at room temperature on a roller platform to allow for the coupling of the capture primer – parasite DNA to the streptavidin-coated magnetic beads. The beads coupled to the parasite DNA were then washed four times, each time with 1 ml of DNA binding buffer. After each wash, the beads coupled to the parasite DNA were separated from the supernatant on a magnetic particle concentrator for 1.5 ml microtubes (DYNAL MPC^®^-E; Dynal AS Oslo Norway). The bound DNA was eluted from the beads by re-suspending in 10 μl of MilliQ water, followed by heating at 80°C for 2 minutes and placed immediately on ice for 2 minutes. Five microlitres of the eluted DNA was used directly as template for PCR.

Amplification of *W. bancrofti *DNA was carried out following the procedure described by Ramzy *et al. *[3]. The PCR products were electrophoresed and visualized in a 2% ethidium bromide-stained agarose gel.

### Field evaluation of developed PCR protocol

The pool-screening protocol was then evaluated against the dissection method in the field. Mosquitoes were caught over two days using HLC in seven Ghanaian villages; Kikam (04° 55' 42"N, 02° 19' 30"W), Ampain (04° 57' 22"N, 02° 24' 06"W), Azulewonu (04° 56' 50"N, 02° 22' 41"W) and Ankobra (05° 26' 53"N, 02° 06' 45"W) in the Axim District, and Brabea (05° 11' 43"N, 01° 13' 46"W) and Abakrampa (05° 14' 39"N, 01° 13' 53"W) in the KEEA District and Moree (05° 07' 12"N, 01° 12' 48"W).

The total mosquitoes caught at each site were divided and part dissected, and part pool-screened for *W. bancrofti *infections. Pool sizes of 30, 40 and 50 mosquitoes were used instead of 100 for statistical considerations and also because not enough numbers were obtained. The infections rates (expressed over 1000 mosquitoes) were estimated using the algorithm of Katholi *et al*. [10].

## Results

### Field sampling methods

A total number of 597 mosquitoes were obtained using the three methods (see Table 1 for the details). Of these, the best collection method was HLC which accounted for roughly 58%, followed by PSC with 41% and LTC with about 0.3% of the total mosquitoes caught. The results also revealed that at the sites where larger numbers of mosquitoes were caught, HLC performed best.

**Table 1:** Number of mosquitoes obtained by the three sampling methods, *LTC*, (Light trap catches) *HLC*(Human landing catches) and *PSC *(Pyrethrum spray catches)

**Study site**	**LTC**	**HLC**	**PSC**	**Total**
Okyereko	2	165	141	308
Atekyedo	0	180	100	280
Gyinginadzie	0	3	6	9
				
Total	2	348	247	597

Generally it was observed that PCR was not very successful with samples that had not been purified with Dynabeads, either there were no amplifications or the results inconsistent especially for the mosquito pools of 50 and above.

**Figure 1:**
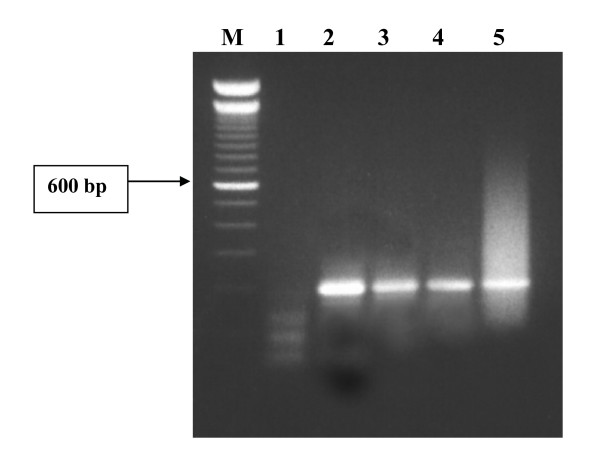
PCR identification of one infective *W. bancrofti *larva in pools of *Anopheles gambiae *s.l. mosquitoes. Gel showing Lanes: M = 100 bp molecular weight marker; 1 = negative control; 2 = positive control; 3–5 = pool sizes of 50, 75 and 100 mosquitoes.

With Dynabead purification however, the successful amplifications of *W. bancrofti *DNA were obtained, with a general tendency of decreasing band intensity with increasing size of mosquito pools (Fig 1).

Consistent positive PCR results were obtained with the DNA samples extracted with Bender buffer and purified with the Dynalbeads and the limit of detection was was 1 L_3 _in 75 mosquitoes. With the DNA extracted and purified with the DNAeasy kit and Dynalbeads respectively, the limit of detection increased to 1 L_3 _in a pool of 100 mosquitoes, and detection of 1 L_3 _in 150 mosquitoes was inconsistent and negative for all the pool sizes of 200.

Table 2 shows the results of evaluating the pool-screening against dissections method. A total of 5802 mosquitoes were caught and 3,042 were dissected and 2,760 were pool screened. No *W. bancrofti*-infected flies were found by both methods at five sites in a total of 3,812 mosquitoes.

**Table 2:** Infection rates of human landing *Anopheles *mosquitoes with *Wuchereria bancrofti *as determined by dissection and PCR pool-screening.

**District/Collection Site**	**Total No. mosquitoes**	**Dissections**			**Pool screen PCR**			
		
		**No. dissected**	**No. infected**	**Infection rate/1000 mosquiotes (CI 95%)**	**No. examined**	**Pool size (No. of Pools)**	**Positive Pools**	Infection rate * (CI 95%)
**Axim District/**								
Kikam	298	118	0	0	180	30 (6)	0	0
Ampain	550	270	0	0	280	40 (7)	0	0
Azulelonu	1080	630	9	14.29 (6.6 – 26.9)	450	30 (15)	5	13.4(4.2 – 31.4)
Ankobra	910	550	11	20.00 (10.0 – 35.5)	360	40 (9)	5	20.1(5.9 – 48.5)
								
**KEEA District/**								
Moree	972	472	0	0	500	50 (10)	0	0
Brebia	856	416	0	0	440	40 (11)	0	0
Abakrampa	1036	586	0	0	550	50 (11)	0	0

* Katholi *et al *(1995)

Infected mosquitoes were however found at 2 sites Azulelonu and Ankobra all in the Axim District. The infection rates determined by dissection were 14.29 and 20.0 per 1000 mosquitoes respectively, and were 13.4 and 20.1 respectively by PCR pool-screening. The observed differences in rates however was not statistically different between the two methods since the 95% CI for both rates overlapped.

## Discussion and Conclusion

Monitoring transmission is an essential component of any LF control programme; for deciding when to stop mass drug administration and also for the certification of elimination of the disease. Monitoring transmission in insects is ideal since mosquitoes may offer a real time estimate of transmission (See Plichart *et al *2006 and Goodman *et al *2003) though the manifestation of mf may be marginally quicker in humans. Very low level microfilaraemia may also not be easy to detect in human populations. Furthermore, the detection of infection in mosquito vectors is an indication that there may be positive individuals in the area.

It has often been argued that for LF programmes if the only required monitoring index of transmission is the infection rate then large numbers of vector mosquitoes are needed irrespective of the collection method. However this study has shown clearly that in terms of numbers and accurate estimation of transmission indices HLC alone can suffice in situations where vector population densities are high. In situations where brute knowledge of transmission (just an indication of the presence of infection) is required and the vector population densities are low both HLC and PSC could be combined. It should be noted however that data from such a combination cannot be used to follow up on changes in transmission. The objective for monitoring will also inform on which method will be most appropriate. For example, xenomonitoring of the infection in human populations requires the use of techniques that capture mosquitoes having a higher chance of containing mf. This will be best achieved by the use gravid traps, indoor resting collections, and knockdown spray catch collections.

Arguments have also been made against dissections as opposed to molecular methods for monitoring transmission. A major constraint of dissections cited in this regard is the large numbers of mosquitoes that need to be processed to find infections in situations of low levels of microfilariaemia; hence the increased interest in PCR based pool screening methods. An important development which favours PCR pool-screening methodology is the algorithm for estimating the infection rate in members of the *Simulium damnosum *complex [10] which potentially can be applied to LF vectors as well [13]. This algorithm was used successfully as a monitoring tool in the Onchocerciasis Control Programme [11].

The consistent detection of one L_3 _larva in a 100 mosquito pool is a significant improvement over previous pool-screening methods developed for the entomological monitoring of LF interventions. This consistency was only observed when the extra step of separating parasite DNA from that of the insect vector using the Dynalbeads method was added. This achievement therefore seems to confirm our intuition that the purity of the parasite DNA template for PCR accounted for the inconsistencies and successes with smaller pool sizes reported by the multi-centre studies [5]. This observation was also made in other studies [13,15]. It seems likely then that as pool sizes increase the insect DNA swamps that of the parasite's DNA making the latter less available in the PCR. This will also explain our lack of consistency with the pool size of 150 even with our improved method. Continued improvement to achieve the detection of 1 in 200 or more pools is necessary to further reduce costs. To achieve this however will need either a highly efficient method of capturing parasite DNA at low concentrations in the extracted DNA solution or as proposed in some quarters utilize real-time PCR [14].

From our experience, 20,000 mosquitoes could be screened with 200 reactions, within a couple of weeks. Most of the time however is spent on the sorting and identification of the mosquito vectors, and not on DNA extraction and the PCR. Moreover once sorted and separated the mosquitoes could be kept dry in pools over silica gels over a period of time until ready to use.

The extra step of DNA purification with the Dynabeads^® ^is the only addition if compared to other DNA isolation methods including the Fischer method [5]. The additional time of this step was an overnight incubation period whilst the extra cost resides in the kit which adds about ¡0.85 US to each reaction. Compared to current methods of identifying 1L_3 _in a pool of 25 mosquitoes, this is very cost effective when the total cost of processing including PCR is examined. The example of 20,000 mosquitoes could be examined in 200 PCR reactions instead of 800 reactions if the pool size is 25.

The usefulness and applicability of the analytical method of Katholi *et al. *[10] for monitoring LF interventions has been attested to by this study in that similar results were obtained for dissection and the pool screening when both were evaluated in the field. However the infections rates obtained by the two methods reveal that they were relatively high at the two affected sites, which will definitely not be the case in instances when intervention has succeeded in reducing circulating parasites to very low levels. We recommend therefore further field evaluation in LF endemic areas presently under MDA and with varying degrees of circulating parasite levels. This will also enable accurate assessment of the cost-benefits of the pool-screening method at various stages of MDA intervention.

## Competing interests

The author(s) declare that they have no competing interests.

## Authors' contributions

All the authors have contributed substantially to this study. DAB and MDW contributed intellectually to the conceptualization, design and initiation of both the study and manuscript preparation. HB and EG carried out the laboratory and field studies. CB advised and contributed to the optimization of the PCR technique and analysis of field data. MAA contributed towards the design, preparation and implementation of the field.
